# Innovative approaches to enhancing tamarind seed germination and phytochemical production through laser irradiation: implications for photodynamic therapy

**DOI:** 10.1007/s10103-025-04527-3

**Published:** 2025-06-20

**Authors:** Manar Hassan, Galal Khamis, Heba El Zorkany, Shaimaa Alexeree

**Affiliations:** 1 National Institute of Laser Enhanced Sciences, Cairo University, Giza, 12613 Egypt; 2 Agricultural Research Centre, Orman, Giza, Egypt

**Keywords:** Germination, He Ne laser, Plant growth, Laser effect, Antimicrobial

## Abstract

*Tamarindus indica*, commonly known as tamarind, is a fruit tree belonging to the Leguminosae (Fabaceae) family, recognized for its traditional medicinal uses. Tamarind leaf extract is rich in antioxidants and anti-inflammatory compounds and possesses antimicrobial and antifungal properties. Despite its benefits, enhancing the germination and seedling quality of tamarind seeds remains a challenge. This study aims to explore the effects of laser irradiation processing on tamarind seeds, examining both the phytochemical changes in seedlings post-irradiation and the biological activities of tamarind leaf extracts before and after helium-neon (He-Ne) laser treatment. Tamarind seeds were irradiated using a red He-Ne laser at 630 nm for 10 min. The experimental design included control groups of non-irradiated seeds for comparative analysis. Following irradiation, various parameters were measured. Additionally, tamarind leaf extracts were prepared for antimicrobial and anticancer activity assessments. Irradiation of tamarind seeds significantly enhanced several growth parameters compared to non-irradiated controls. Specifically, there was an increase in germination percentage, dry weight of plant material, total protein content, total flavonoids, total phenols, and 2,2-Diphenyl-1-picrylhydrazyl (DPPH) activity in seedlings from irradiated seeds. The laser-processed extracts exhibited significant antibacterial activity against both gram-positive and gram-negative bacteria. Furthermore, photodynamic treatment demonstrated that laser processing effectively suppressed the growth of human cancer cell line (A549) cancer cells at lower concentrations compared to untreated extracts. The study concludes that laser irradiation is an effective method for enhancing the germination and seedling quality of *Tamarindus indica*. Additionally, it positively influences the phytochemical properties of tamarind leaf extracts as a bio-drug, enhancing their biological activities. These findings suggest that laser irradiation not only improves seed germination and crop yield but also enhances the phytochemical profile of tamarind leaves, potentially increasing their therapeutic efficacy.

## Introduction

Diets based on plants have received a lot of attention since they are significant sources of phytonutrients and have been demonstrated to have protective effects against many diseases [1, 2]. *Tamarindus indica (T. indica)*, commonly known as tamarind, is a remarkable evergreen tree belonging to the Fabaceae family, native to tropical Africa but extensively cultivated across tropical and subtropical regions, particularly in India and Central America. *T. indica* is among the most nutritious plants and is thought to represent a vast storehouse of several phytochemicals, including phenolic compounds, unsaturated fatty acids, amino acids, carbohydrates, vitamins, and minerals. The tamarind tree produces distinctive brown, pod-like fruits that contain a sweet and tangy pulp and can grow up to 24 m tall [3].

In addition to its culinary use, tamarind pulp is prized for its therapeutic qualities, which may include anti-inflammatory and antioxidant effects [4]. Because it contains a high concentration of proteins together with various other necessary phenolic compounds, malic acid, glycosides, xylose, glucose, galactose, and amino acids, these play a significant role in the development of powerful and capable muscles [5–7]. Moreover, *T. indica* is also an excellent source of minerals, including high concentrations of calcium, copper, iron, cadmium, manganese, arsenic, sodium, magnesium, potassium, phosphorus, zinc, and lead, as well as fatty acids and carbohydrates that provide energy [8, 9]. The leaves of *T. indica* are high in fatty acids, lipids, flavonoids, and vitamins. These tamarind plant leaves have tremendous potential as a source of effective medicinal products [10].

Additionally, tamarind seed coat extract effectively inhibits reactive oxygen species (ROS) in human skin fibroblast (CCD-1064Sk) cells, increasing glutathione and antioxidative enzymes like glutathione peroxidase-1 (GPx), superoxide dismutase (SOD), and catalase activity [11]. *T. indica* leaf powder treatment for four weeks improved lipid and carbohydrate profiles in albino rats, decreasing glucose, peroxidation, cholesterol excretion, glycogen levels, and antioxidant profiles in renal and hepatic tissues [12, 13]. Because of the presence of phytonutrients and the total phenolic content, the pulp of tamarind is rich in many phytonutrients that function as effective dietary antioxidants [2]. So, increasing the levels of bioactive compounds in tamarind is in great demand for improving their capability as food with high nutritional values and biological activities.

The potential of laser technology to improve phytochemical production and plant health in general has attracted much interest in its application in plant biology and agriculture [14]. As a physical stimulator, laser irradiation can stimulate various physiological and biochemical processes in plants, significantly altering their metabolic profiles. This observable phenomenon is especially important when it comes to increasing the quantity and quality of bioactive chemicals, which are necessary for both therapeutic and nutritional applications. According to studies, laser light can improve photosynthetic efficiency, increase biomass accumulation, and improve seed germination, all of which have a positive impact on plant growth and development [15, 16]. For example, research has demonstrated that exposure to select laser light wavelengths, such as He-Ne lasers, can increase the amount of chlorophyll and promote cell division, both of which eventually lead to greater plant vigor. Furthermore, some plant species have shown improved mycorrhizal colonization following laser treatment, which further enhances nutrient intake and growth. Additionally, laser irradiation influences secondary metabolites such as flavonoids, phenolic compounds, and essential oils. These phytochemicals play vital roles in plant protection mechanisms and possess various health benefits for humans [17–20].

To the best of our knowledge, there is little information about using laser treatment to improve the levels of bioactive compounds and germination rate in tamarind plants. Consequently, in our research, we hypothesized that the tamarind seeds pre-illumination using a He-Ne laser with a wavelength of 630 nm would increase the germination rate and the antioxidant capacity. The production of phytochemicals will ultimately rise, enhancing the biological activity of tamarind leaf extracts as antibacterial and anticancer agents.

## Materials and methods

### Materials

Using a Milli-Q water purification system, deionized water was used to prepare all the reactive material solutions. Aqua regia (hydrochloric acid: nitric acid (HCl: HNO₃) = 3:1 (v/v)) was used to wash each glass, followed by Milli-Q water.

### Extraction method

We primarily utilized Soxhlet extraction due to its efficiency in extracting active constituents from plant materials. In this method, tamarind leaves were air-dried at a controlled temperature to reduce moisture content while preserving active compounds. The dried leaves are finely ground into a powder to increase surface area, enhancing solvent penetration. Powdered leaves are soaked in methanol, typically in a 1:10 ratio of plant material to solvent, for 24–48 h at room temperature or with gentle shaking. After soaking, the solution is filtered to remove solids, and methanol is evaporated using a rotary evaporator to yield a concentrated extract, which is then stored at low temperatures to preserve its chemical integrity before further testing [21].

### Experimental design

#### Plants experiment

##### Seed collection

Seeds of *Tamarindus indica* have been collected from a trusted local market in Aswan, Egypt. Typically, healthy, dark brown, hard, shiny, uniform-sized, and uniform-colored seeds were chosen. The collected plant pods were washed with tap water, followed by distilled water to obtain seeds, and dried in the shade at room temperature for one day before laser pre-illumination.

#### Experimental design of laser pre-illumination of seeds

##### Seed treatment and planting conditions

After initial selection, tamarind seeds were divided into two groups at random. Each group contains 30 seeds. As a control, the first group was not exposed to any radiation. The second group, called the experimental group, was exposed to laser pre-illumination using a helium-neon (He-Ne) laser (model: Laser II, DMC Equipment Ltd.; wavelength: 630 nm; power: 20 mW). The laser beam was pointed perpendicular to the seed surface, and each seed was exposed to it separately for ten minutes. Following treatment, all seeds were planted in 15 cm plastic pots filled with good drainage loam soil with a pH of 6.0–6.5. The pots were kept in natural summer settings, which offered the ideal growing circumstances for tamarind seedlings. Regular irrigation was done, and during the second week after planting, weekly applications of nitrogen, phosphorus, and potassium (NPK) fertilizer (10:10:10) were made. During the course of the experiment, manual weed removal was done as needed.

##### Germination monitoring and sample collection

Three weeks following planting, the first emergence was noted, and germination rates were tracked. In comparison to the control group, the laser-treated group’s germination percentage was noticeably greater. After two months, the tamarind seedlings were taken out for chemical examination.

### Characterization

#### Gas Chromatography-Mass spectrometry (GC-MS) analysis

For the determination of leaf extract samples, GC–MS analysis was performed. The test samples’ active components were separated using a closed column made of fused silica and helium gas as the mobile phase. A single µL aliquot of every extract was introduced into the GC-MS device. The column’s initial temperature was set to 60 °C. The components were identified by comparing the components’ mass spectra with those of the NIST mass spectral collection [22, 23].

#### Determination of the total phenolic content

The total phenolic content in extracts was determined using a modified Folin-Ciocalteu technique, which involves agitating 100 µL of each extract with 500 µL of Folin-Ciocalteu reagent and deionized water [24].

#### Determination of total flavonoids

Total flavonoid content was determined by a colorimetric method. The 80/100 g methanolic extract was diluted with distilled water, sodium nitrite (NaNO₂) solution, aluminum chloride hexahydrate (AlCl₃ 6 H₂O) solution, sodium hydroxide (NaOH), and distilled water. The mixture was then mixed, and absorbance was measured at 510 nm using a Jasco UV-530 spectrophotometer, expressing the results as mg of catechin equivalents [25].

#### 2,2-Diphenyl-1-Picrylhydrazyl (DPPH) radical scavenging activity

Trolox stock standard solution in methanol was prepared in various concentrations, and a DPPH free radical assay was conducted using a 0.3 mg/mL methanol (MeOH) sample [26].

#### Protein extraction

The leaves were frozen, ground, and incubated with 1% NP-40 extraction buffer. Samples were centrifuged twice, and the supernatant was collected for protein fractionation. The proteins were then subjected to poly(ethylene glycol) (PEG) fractionation [27].

#### UV-Visible spectroscopy analysis

The spectral absorption of the control and irradiated groups was detected by UV–Vis spectroscopy. For absorption measurements, aliquots (200 µL) of the prepared extracts were transferred to 96-well plates, and absorptions were recorded within a scan range of 300–750 nm using UV-Visible power wave microplate reader spectroscopy (BioTech, USA).

### Bacterial experiment

There were two common bacterial suspensions employed. Gram-negative (-Ve) bacteria, Escherichia coli O157 (*E. coli O157*), which are represented by the first suspension. As an example of a Gram-positive (+ Ve) bacterium, Bacillus subtilis (*B. subtilis*) is the second suspension. The bacteria were cultured aerobically at 37 °C for the entire night on nutritional agar. The following procedures were used to prepare the standard suspension of the *B. subtilis* and *E. coli O157* strains, which contained 10^9^/mL live cells: (1) Each species of the bacterium was obtained as a single colony from an agar plate and injected into Luria Bertani (LB) broth media. (2) The inoculated broth is incubated at 37 °C for 24 h. (3) A UV-Visible power wave microplate spectrophotometer (BioTech, Vermont, USA) was used to measure the number of viable cells in each bacterial culture to an optical density (OD_600_ nm) (1 × 10^9^ cells/mL). (4) To initiate the inoculum of around 10^6^ colony-forming units/mL, the bacterial suspension was diluted with LB broth media. Agar plate counts revealed the locations of these colonies [28].

### Cell culture experiment

A549 cells were purchased from “The Egyptian Organization for Biological Products and Vaccines (VACSERA)” and were cultured in Roswell Park Memorial Institute (RPMI-1640) media supplemented with 10% Fetal Bovine Serum (FBS), 2 mM L-glutamine, 0.01 mL penicillin, and 100 mg/mL streptomycin (Lonza, Belgium). The cells were cultured in an environment that was humidified with 5% carbon dioxide (CO₂) at a consistent temperature of 37 °C.

### Light source used in photodynamic therapy

A light-emitting diode (LED) with a red wavelength was used to excite the samples as a source of irradiation with homogeneous and stable irradiation. LED (LED Edixeon, Edison Opto Corporation, New Taipei City, Taiwan) produces a light emission at the red wavelength of 650 nm with a power density of 50 mW. The irradiances (energy fluencies) of the red LED array tested in this study were 12 J/cm², using a PM100D power/energy meter (Thorlabs, Inc., Newton, NJ). This was adapted by regulating the distance between the bacterial cells and the LED array aperture.

### Experiment of photodynamic therapy (PDT) in vitro

The A549 cells were displayed to L and U with three different concentrations: 0.0625, 0.03125, and 0.0156 mg/mL from each sample. Every concentration was prepared in three replicates. 150 µL of each A549 was seeded in a 96-well microtiter plate with a flat bottom. To estimate the effect of photoinactivation, the experiment is divided into three groups. These groups are categorized as (1) negative control group. 2) Dark toxicity group: three distinct L and U doses are incubated with A549. 3) The PDT group, A549, was exposed to varying doses of the chemicals L and U for 30 min at 37 °C in a dark environment. A red LED was used to irradiate the PDT experiment group at a dosage of 12 J/cm². The constant temperature conditions in 96-well plates were provided. Samples were analyzed at a temperature of 37 °C, shaking between measurements, and data recording after 24 h. Survival was calculated from the last point in the growth curves, related to the control value [29].

### Statistical analysis

The mean of three treatments ± standard deviation (SD) was used to express cell survival, and GraphPad Prism 7.00 was used to determine the significance of any changes from the control. The two-way ANOVA test was used to determine statistical significance (*P* < 0.05) for examining the effects of test material concentration and laser irradiation period.

## Results and discussion

In the current investigation, tamarind seeds were pre-illuminated using a He-Ne laser with a wavelength of 630 nm for 10 min, which increased the germination rate and the antioxidant capacity, resulting in improved biological activities of tamarind leaf extracts as antibacterial and anticancer after laser processing compared to the non-irradiated tamarind seeds.

### GC/MS analysis of the leaf extract of Tamarindus indica

Table 1 indicates a traditional GC-MS analysis output of a methanolic leaf extract of *T. indica.* Analysis represents retention times (Rt), compound identifications, molecular formulas, and quantitative data (area percentages and peak areas). The retention time, ranging from 4.53 to 21.68 min, provides crucial qualitative information that has been used to identify specific compounds. Under consistent analytical conditions, these retention times serve as characteristic identifiers for each compound, allowing for reliable identification when compared against standard samples. Percentages of peak area represent the relative abundance of each compound in the sample. The compound 3-O-methyl-d-glucose dominates at 22.28%, which indicates that this glucose derivative constitutes nearly a quarter of the detected compounds. This may be related to the fact that it is a primary bioactive component of the analyzed material [30].

Additionally, the table highlights a spectrum of bioactive compounds with significant health-promoting properties. Fatty acids (such as palmitoleic acid, lauric acid, and linoleic acid), sterols (like α-sitosterol and β-sitosterol), and other organic molecules collectively contribute to antimicrobial, antioxidant, anti-inflammatory, metabolic, and antitumor activities. These findings underscore the importance of such bioactive materials in both nutritional and therapeutic contexts, supporting their continued study and application in health sciences [30, 31].

The bioactive compounds identified, particularly fatty acids and sterols, exhibit a wide spectrum of significant physiological and pharmacological activities. Palmitoleic acid, a monounsaturated fatty acid, is increasingly recognized for its role in modulating lipid metabolism, insulin sensitivity, and inflammatory responses, thereby influencing metabolic health and diseases such as diabetes, cardiovascular disorders, and cancer. Similarly, lauric acid and its derivatives demonstrate potent antimicrobial effects by disrupting microbial membranes, alongside antitumor, hepatoprotective, and neuroprotective properties mediated through antioxidant and cell signaling pathways. Other fatty acids, including linoleic acid and vaccenic acid, contribute to maintaining cell membrane integrity and exert anti-inflammatory and cardioprotective effects. Additionally, pentadecanoic acid and 9-octadecenoic acid esters play important roles in metabolic regulation and inflammation control, supporting overall lipid homeostasis [32, 33].

Sterols such as α-sitosterol further complement these bioactivities by lowering cholesterol absorption and exhibiting anti-inflammatory, anticancer, and immunomodulatory effects, positioning them as valuable nutraceuticals. Other compounds identified, including cyclopropane fatty acid derivatives and bile acid analogs like ethyl iso-allocholate, suggest additional antimicrobial and metabolic regulatory functions. Moreover, molecules like 3-oxo-20-methyl-11,3-hydroxyconanine-1,4-diene, though less characterized, share structural similarities with known antitumor and antioxidant agents, indicating potential therapeutic relevance. Collectively, these bioactive materials demonstrate multifaceted roles in antimicrobial defense, oxidative stress reduction, metabolic modulation, and cancer prevention, underscoring their importance in health promotion and the development of novel therapeutic strategies [34–36].

**Table 1:**
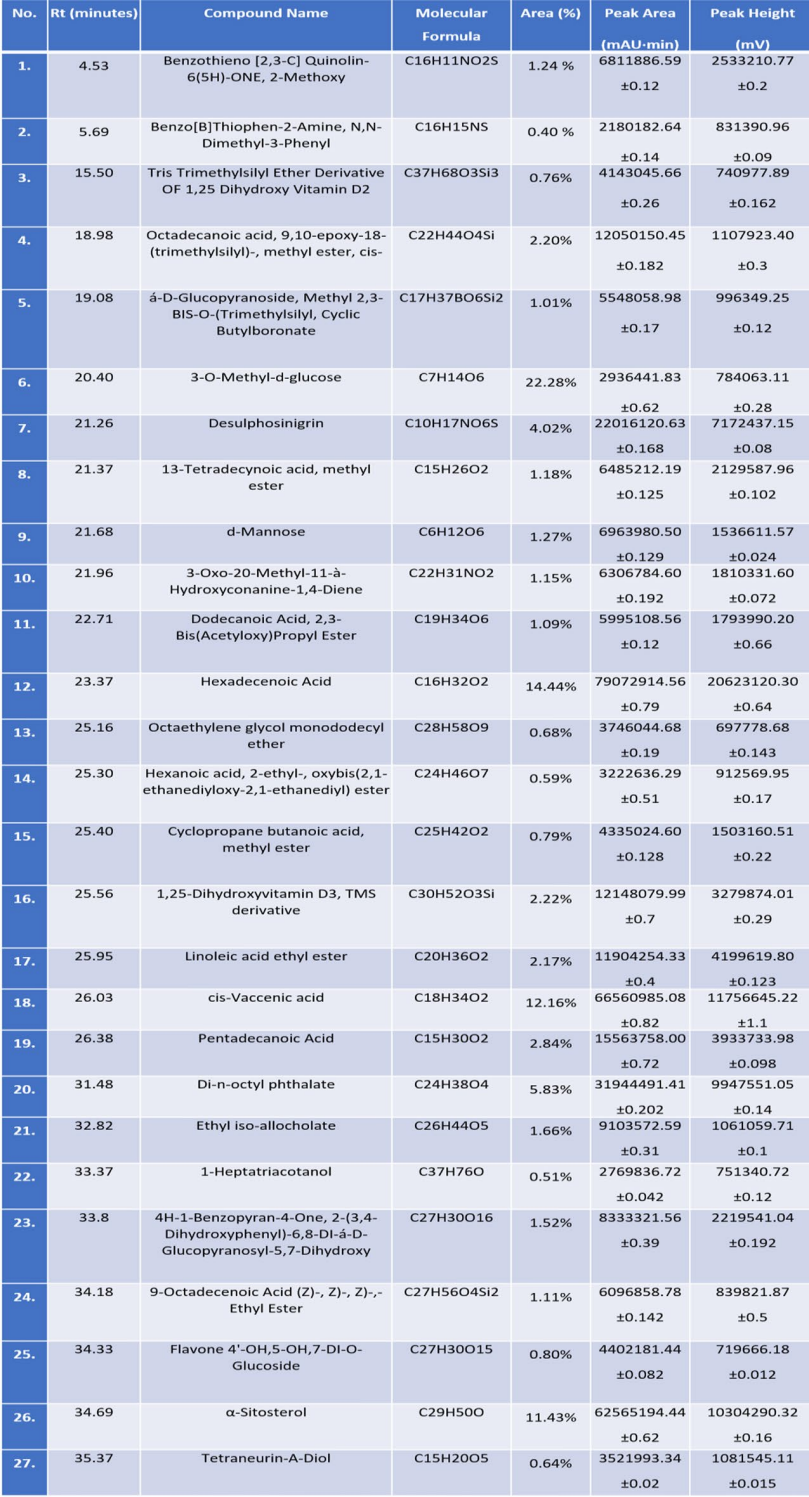
Indicates GC/MS analysis of the leaf extract of *Tamarindus indica*. Data is represented by the means of at least 3 replicates ± standard deviations

### Effect of laser treatment on the growth, nutritive values, and antioxidant capacity of T. indica

The physiological and biochemical properties of tamarind seedlings were significantly impacted by laser processing (Table 2). The laser-treated group’s germination rate increased from 53.33% in the untreated group to 66.67% (*p* < 0.01), indicating that the enhanced metabolic activation and seed vigor were caused by photobiomodulation effects. Root length dropped marginally from **17.70** cm to 15.70 cm, but the difference was not statistically significant (*p* > 0.05). On the other hand, shoot height increased significantly from 15.90 cm to 19.90 cm (*p* < 0.01), indicating better shoot development that may have been caused by different hormonal signals during laser exposure.

As a result of increased biomass accumulation and metabolic activity in treated plants, dry weight increased from 1.53 g to 2.08 g (*p* < 0.05). Total protein concentration increased significantly from 0.34 ± 0.02 to 0.38 ± 0.002 mg/mL (*p* < 0.001) in accordance with this, suggesting better protein synthesis or nitrogen assimilation.

The total amount of flavonoids significantly decreased in the laser processing group when compared to the untreated (control) group. After laser treatment, the concentration dropped from 9.18 ± 1.13 µg/mg in untreated samples to 4.46 ± 0.13 µg/mg in laser-treated samples (p < 0.01). This decrease raises the possibility that laser irradiation may suppress the manufacture of flavonoids or reroute metabolic precursors to other phenolic molecules. Additionally, the decrease might be the result of a metabolic shift toward alternative phenolic molecules, even in the face of increased growth and antioxidant activity. The observed reduction here may reflect such a metabolic shift rather than a general suppression of secondary metabolism. This was corroborated by the fact that total phenolic acids rose from 52.5 ± 2.14 to 62.07 ± 3.57 µg/mg (*p* < 0.01), indicating a relocation within the phenylpropanoid pathway. In one instance, DPPH scavenging activity showed a substantial increase from 194.94 ± 6.90 to 263.53 ± 1.53 (*p* < 0.001), suggesting a greater antioxidant capacity [37].

Phytochemical synthesis observed an increase in particularly phenolic acids and total antioxidant capacity, which can be attributed to the plant’s physiological response to laser-induced abiotic stress. In plant tissues, laser irradiation is known to function as a low-dose stressor that can activate signaling pathways linked to defense. Frequently, this stimulation results in the upregulation of secondary metabolite biosynthesis, including phenolics and other antioxidant compounds, as part of a protective adaptation [38]. Specifically, exposure to red light wavelengths (e.g., He-Ne laser) can enhance the activity of key enzymes such as phenylalanine ammonia-lyase (PAL), which catalyzes the first committed step in phenolic compound biosynthesis. The resulting increase in phenolic content contributes to improved reactive oxygen species (ROS) scavenging capacity, as evidenced by higher antioxidant activity post-treatment. Therefore, the laser-induced enhancement of phytochemicals likely reflects a photobiomodulatory effect, promoting metabolic reprogramming to improve cellular defense and adaptation under mild stress conditions [39, 40].

In agreement with our findings, Mousa et al. (2022) reported that laser treatment for seeds of *B. aegyptiaca* at 200 mW/4 min induced the yield percentage, total phenolic and flavonoid contents, and DPPH compared to untreated *B. aegyptiaca* seeds [41]. This increased activity is probably the result of particular antioxidant molecules being synthesized more frequently in response to mild oxidative stress brought on by laser exposure. Furthermore, laser-treated common buckwheat (CBW) and tartary buckwheat (TBW) sprouts were tested for the presence of health-promoting minerals, metabolites, and enzymes, as well as for their antioxidant and anti-inflammatory properties (He–Ne laser, 633 nm, 5 mW). After CBW and/or TBW sprouts were exposed to laser light, over 35 parameters of 49 specified minerals, vitamins, pigments, and antioxidants were considerably elevated. Additionally, laser light increased the anti-inflammatory and antioxidant properties by blocking the actions of lipoxygenase and cyclooxygenase 2, especially in TBW sprouts [42].

Irradiation light enhanced snap bean growth, yield, and biochemical characteristics in earlier studies. Moreover, the same enhancement appeared in the anatomical structure of the leaf and gene expression stability [43]. The obtained results agreed with other work done on mustard, cauliflower, and turnip plants when they were exposed to He–Ne radiation, showing an increase in biomass, leaf count, and overall fresh weight. The red light accelerates the rhythm of plant growth, increasing root growth, which increases plant height, another sign of plant growth. Red illumination also increases biomass and encourages plants’ vertical development. As a result, comparable changes were seen in the leaf’s length and width by this principle [44, 45].

It has been suggested that laser light promotes plant growth and seed germination. A specific wavelength of laser light that is absorbed by phytochromes tends to increase the internal energy of seeds by converting light energy into chemical energy [46]. This, in turn, increases cell pumping and improves the electro potential of biomembranes. Therefore, the energy generated is employed in stimulating the rate of germination, eliciting thermodynamic characteristics, and triggering physiological and biochemical mechanisms. In addition, this energy promotes the activities of enzymes like protease and amylase, which enhance plant development and yields; both processes speed up cell division [47].

**Table 2:**
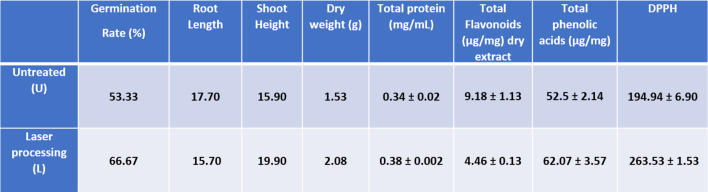
Effect of laser processing on seed germination rate, seedling dry weight, total protein, total flavonoids, total phenolics, and DPPH compared to the untreated. Data is represented by the means of at least 3 replicates ± standard deviations

### Effect of laser processing on the absorption wavelength of T. indica

**Fig. 1:**
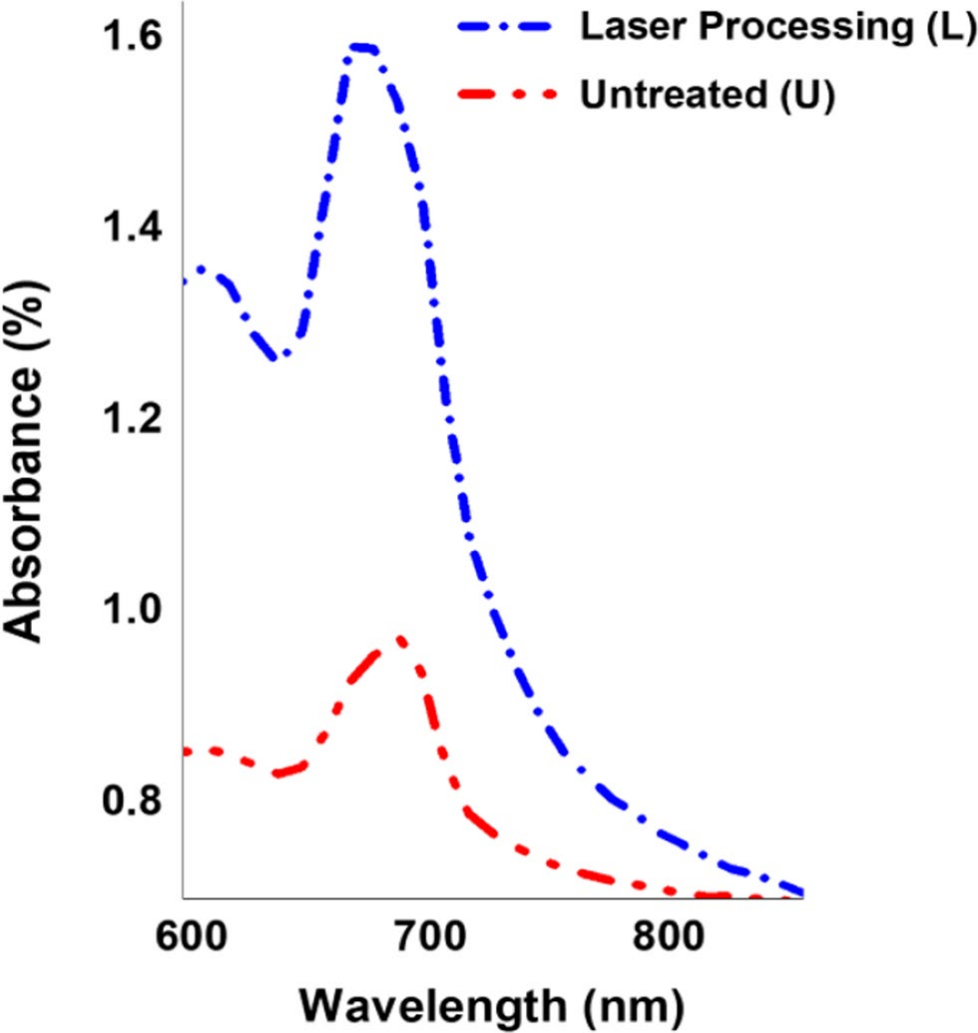
Shows the absorption wavelength of tamarind in the visible region before laser treatment (untreated, U) and after laser processing (L)

Ultraviolet-visible spectroscopy, as seen in Fig. 1, displays the schematic design for the absorbance that was determined by using UV-Visible spectroscopy to analyze an extract of leaves from *T. indica.* Research is still being done to determine how laser irradiation affects plants’ absorption wavelength. Different laser wavelengths’ effects on plant growth and development have been the subject of several investigations. Here, the He-Ne laser significantly increased the absorption wavelength of tamarind leaf extract compared to the corresponding control plants. This is because laser irradiation can increase the morphological parameters and product component of *T. indica* and induce germination by breaking the dormant period of seeds [48]. Moreover, these results imply that laser irradiation at specific wavelengths, 630–650 nm, can significantly affect plant physiology and crop yield. However, the precise impacts on the plant’s absorption wavelength may differ based on the plant species and the experimental setup. Additionally, laser irradiation can modify both living and non-living materials with submicron accuracy without physical touch. It is an important tool for the procedure known as photoinjection, which permeabilizes cellular membranes to transport molecules [49]. Additionally, the absorption spectrum of *T. indica* shows that the present compounds have maximum absorption in the range of 600–700 nm with a peak absorption of 665 nm. Delightfully, this peak is in the region of red and near-infrared, where it is explained by the chloroplast, an essential part of the leaf’s photosynthetic process.

### Antimicrobial activity of T. indica

In the present study, the antimicrobial activity was screened for laser-processed tamarind and untreated tamarind separately on the Gram-positive bacteria (*B. subtilis*) and the Gram-negative bacteria (*E. coli O157*) by the turbidimetric method using a microplate assay. Deionized distilled water (d.d. H₂O) was used as a solvent for the two samples.

**Fig. 2:**
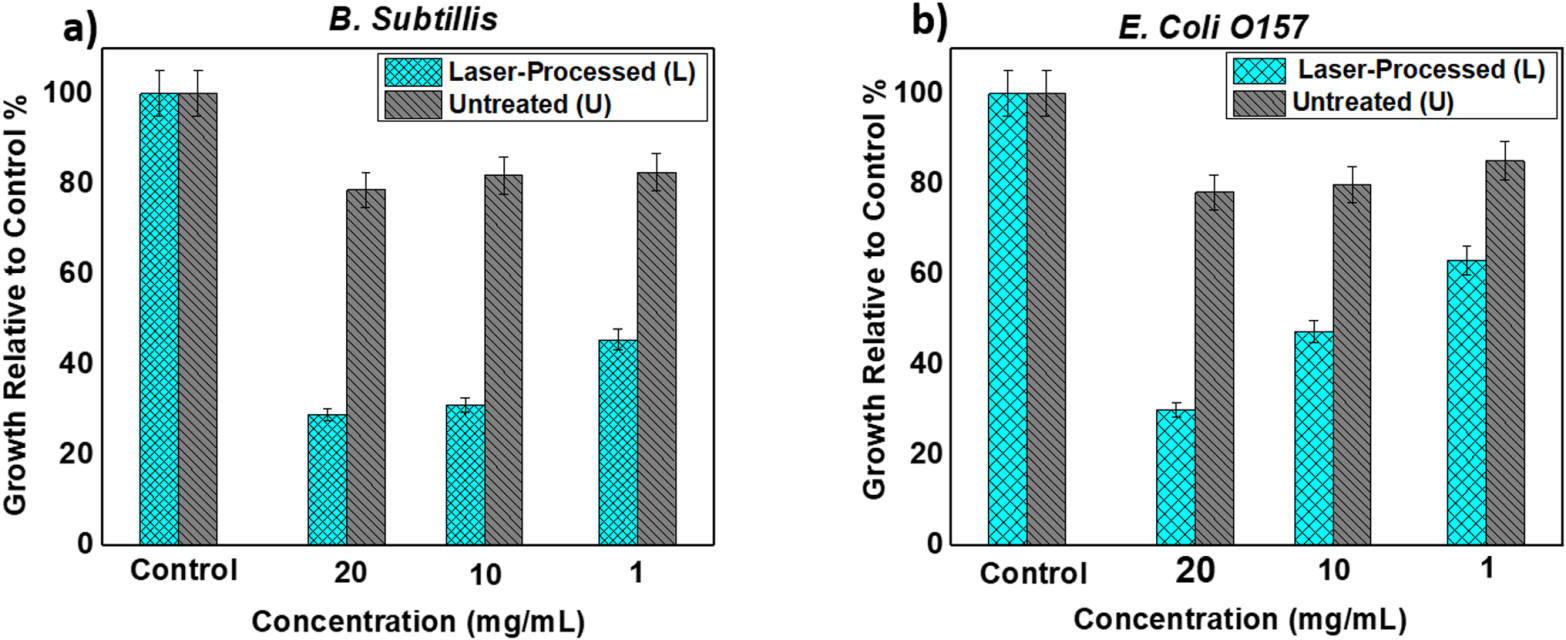
Shows the antimicrobial activity of laser-processed tamarind extract (L) and untreated tamarind extract (U) for both gram-positive (**a**) *B. subtilis* bacteria and gram-negative (**b**) *E. coli O157* bacteria

Figure 2a indicates the dark toxicity of laser-processed tamarind (L) and untreated tamarind (U) at three different concentrations of *B. subtilis* relative to the negative control. Negative control represents the bacterial strain without any addition. The results showed that The inhibitory effect of the laser-processed extract exhibits a significantly superior compared to the untreated extract across all tested concentrations (1, 10, and 20 mg/mL). Laser-processed tamarind extract has a significant toxic effect on *B. subtilis* bacteria than untreated tamarind extract compared to the control. According to experimental findings, phenols are chemicals that work against *B. subtilis* cultures but not against other organisms, based on experimental results [50]. Moreover, in the case of laser-processed tamarind, the yield of phenolics from dry extract was more than untreated tamarind dry extract, as mentioned above. Furthermore, presence of lauric acid and palmitoleic acid in *T. indica* may be responsible for damaging the microorganisms membranes, conferring broad-spectrum antimicrobial efficacy. Additionally, we observed that low concentration has also a significant effective in the laser processing tamarind extract. The laser exposure likely induces chemical modifications or promotes the release of these phytochemicals, thereby intensifying the extract’s ability to inhibit bacterial growth. For instance, cyanobacterial extracts directly exposed to laser irradiation showed significantly larger inhibition zones compared to untreated controls, confirming the laser’s role in enhancing antimicrobial potency [51, 52].

Figure 2b also shows the antimicrobial action of the two extracts at three different concentrations (1, 10, and 20 mg/mL). Laser-processed (L) exhibits significantly greater efficacy than the untreated (U) extract. This may be attributed to an extract increase in a variety of bioactive components, for example tannins, saponins, terpenoids, glycosides, anthraquinones [10]. There are numerous potential explanations for this, including disruption of membranes, elevated production of reactive oxygen species (ROS), and disruption of bacterial metabolism and reducing sugars that are responsible for their antimicrobial activity. The broad-spectrum activity against diverse bacterial strains underscores the potential application of laser-processed extracts as natural preservatives or antimicrobial agents in food safety and medical fields, providing an alternative to synthetic chemicals with reduced toxicity and environmental impact [53, 54].

In last, Laser-processed (L) tamarind extract demonstrates greater effectiveness against Gram-positive bacteria than Gram-negative bacteria related to the control. This difference in susceptibility is commonly attributed to the structural variations in the bacterial cell walls. Gram-positive bacteria possess a thick peptidoglycan layer that is more accessible to antimicrobial agents, whereas Gram-negative bacteria have an additional outer membrane that acts as a barrier, limiting drug penetration and reducing susceptibility [55].

### Photodynamic therapy in vitro

Photodynamic therapy (PDT) is a noninvasive therapeutic method used for cancer therapy. In PDT, malignant tumors are treated by inducing apoptosis or necrosis of cancer cells. PDT, also known as photochemotherapy, combines light, oxygen, and a photoactive medication known as a photosensitizer. In photochemotherapy, a certain wavelength of light excites the photosensitizer, which subsequently releases its energy into oxygen to produce reactive oxygen species **(ROS)** that cause either necrotic or apoptotic cell death [55, 56].

**Fig. 3:**
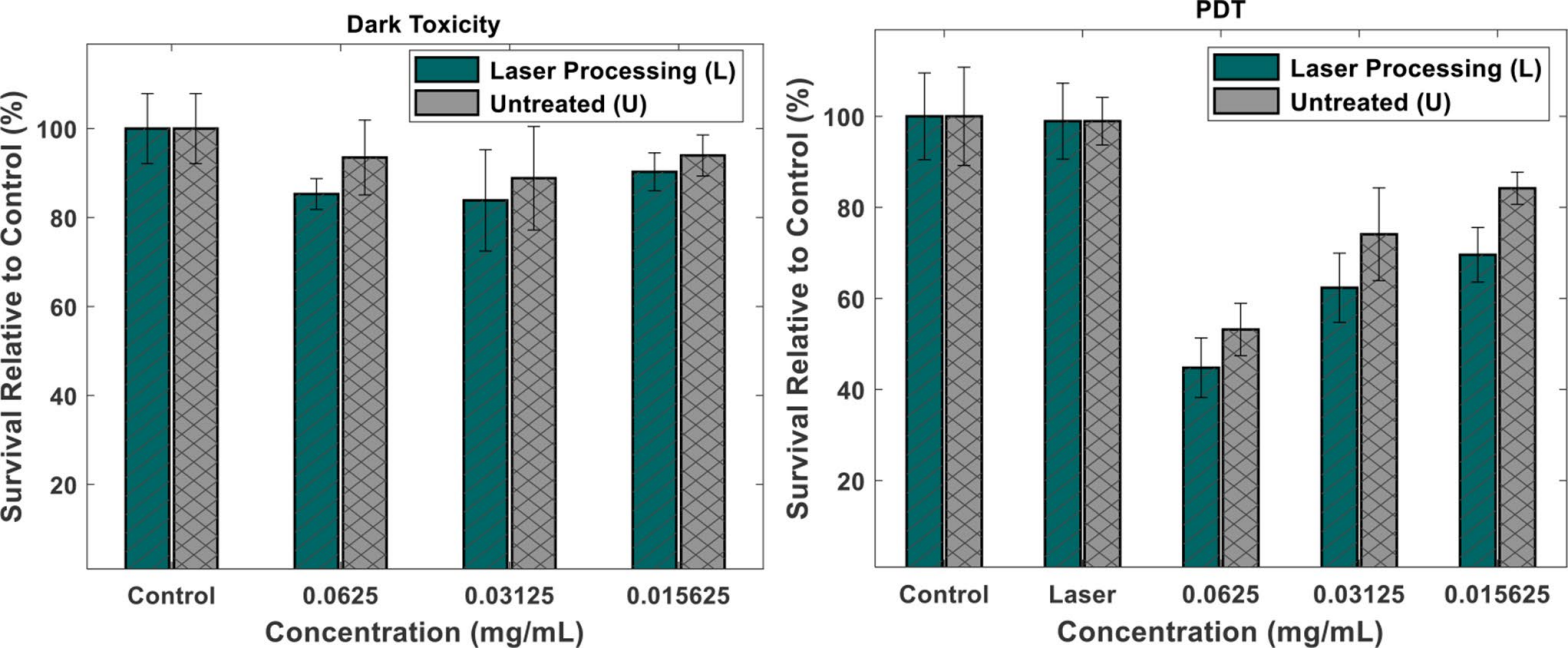
Shows the anticancer activity of laser-processing tamarind extract (L) and untreated tamarind extract (U) for A549 cancer cells in the (**A**) Dark toxicity and (**B**) Photodynamic therapy (PDT)

In this research the viability of the A549 human lung cancer cell line was assessed in the dark conditions, and after the photodynamic effect, the data are shown in Fig. 3. The cytotoxic assay of the leaf extract of *T. indica* was evaluated in the A549 cell using the 3-(4,5-dimethylthiazol-2-yl)-2,5-diphenyl-2 H-tetrazolium bromide (MTT) reduction assay. Following treatment with different doses of the leaf extract in the dark did not significantly inhibit A549 cells (*P* > 0.05), as a low reduction in cell viability was observed in Fig. 3a. On the other hand, the PDT experiment results revealed that laser-processed *T. indica* (L) has significantly (*P* < 0.05) succeeded in suppressing the growth of A549, as illustrated in Fig. 3b.

A red light-emitting diode (LED) at a dose of 12 J/cm² is used as a source of light irradiation. This wavelength is known for its ability to penetrate biological tissues and activate photosensitive compounds. In this study, *Tamarindus indica* was evaluated for the first time as a natural photosensitizer, tested at three different concentrations to assess dose-dependent effects. The rationale for exploring *T. indica* lies in its rich phytochemical profile, including polyphenols, flavonoids, and phenolic acids, which are known to exhibit photoactivity under red light exposure.

Untreated *T. indica* extract (U) demonstrated inherent cytotoxicity against A549 human lung carcinoma cells, indicating the presence of bioactive compounds with potential anticancer effects. Upon laser processing (L)—a technique shown to enhance the biosynthesis of secondary metabolites—the cytotoxicity of *T. indica* was significantly increased (p < 0.05), even at lower dosages. This enhancement is likely due to the increased concentration of photosensitive phytochemicals, which, upon red-light activation, may generate reactive oxygen species (ROS), leading to oxidative damage and apoptosis in cancer cells. This mechanism aligns with the principles of photodynamic therapy (PDT), where light-activated agents induce selective cytotoxicity in malignant cells while minimizing damage to surrounding healthy tissues [57].

According to these results, *T. indica* extract treated with a laser may be used as a novel plant-derived photosensitizer with potential uses in PDT and cancer treatment. To clarify the exact biochemical mechanisms at play and evaluate the safety and specificity of this strategy in vivo animals, more research is necessary.

The action mechanism of this result may be related to the presence of strong anticancer components as obtained from the GC-MS. These chemical components include β-sitosterol (BS), monounsaturated fatty acids (MUFAs), linoleic acid, ricinoleic acid, phytanic acid, and testolactone. These compounds may act as photosensitizers. These compounds are activated through red LED irradiation. This is in the red and near-infrared spectrum and is associated with the chloroplast, an essential part of the photosynthesis reaction in plants. Thus, it is conceivable that photodynamic damage will transpire close to the PS’s intracellular site. PDT can cause cell death by the three primary morphologies of cell death: necrotic, apoptotic, and autophagy-associated. In general, it is acknowledged that when cells are exposed to PDT in vitro, apoptosis is the primary mode of cell death [58].

## Conclusion

*T. indica* is an inexpensive and widely accessible plant. It is a great source of vitamins, phytochemicals, and vital amino acids. Through laser irradiation processing, this work aims to improve the germination of tamarind seeds and the quality of the seedlings while investigating phytochemical changes in the irradiated seedlings. Furthermore, it investigates the antibacterial and anticancer properties of tamarind leaf extracts both before and after He-Ne laser processing. The results showed that the germination percentage, dry weight of plant material (g), total protein (mg/mL), total flavonoids (µg RE/mg dry extract), total phenols, and DPPH of the seedlings grown from the irradiated seeds (10 min irradiation) were higher than those of the untreated seeds (non-irradiated). In addition, studying the biological activities of tamarind leaf extracts as antimicrobials before and after He-Ne laser processing. Remarkably, the data show that, in comparison to the control, the laser-processed extract has a significantly harmful effect on both gram-positive and gram-negative bacteria. The PDT experiment showed that laser processing *T. indica* (L) effectively suppresses A549 cell growth at a lower concentration than untreated *T. indica* (U) in the presence of light.

## Data Availability

No datasets were generated or analysed during the current study.

## References

[1] Almuhayawi SM, Almuhayawi MS, Al Jaouni SK, Selim S, Hassan AHA (2021) Effect of laser light on growth, physiology, accumulation of phytochemicals, and biological activities of sprouts of three Brassica cultivars. J Agric Food Chem 69:22, 6240–6250 34033484 10.1021/acs.jafc.1c01550

[2] Martinello F, Soares SM, Franco JJ, Santos AC, Sugohara A, Garcia SB, Uyemura SA (2006) Hypolipemic and antioxidant activities from Tamarindus indica L. pulp fruit extract in hypercholesterolemic hamsters. Food Chem Toxicol 44:810–818 16330140 10.1016/j.fct.2005.10.011

[3] Kuru P (2014) Tamarindus indica and its health-related effectivity. Pac J Trop Biomed 4:676–681

[4] Razali N, Jonit M, Ariffin S, Ramli A, N. S., Abdul AA (2015) Polyphenols from the extract and fraction of T. indica seeds protected HepG2 cells against oxidative stress. BMC Complement Altern Med 15:438 26683054 10.1186/s12906-015-0963-2 PMC4683930

[5] Bursal E, Köksal E, Gülçin İ, Bilsel G, Gören AC (2013) Antioxidant activity and polyphenol content of Cherry stem (Cerasus avium L.) determined by LC–MS/MS. Food Res Int 51(1):66–74

[6] Valdes L, Cuervo A, Salazar N, Ruas-Madiedo P, Gueimonde M, González S (2015) Relationship between phenolic compounds from diet and microbiota: impact on human health. Food Function 6:2424–2439 26068710 10.1039/c5fo00322a

[7] Arshad MS, Imran M, Ahmed A, Sohaib M, Ullah A, Nisa m, Hina G, Khalid W, Rehanal H (2019) Tamarind: A diet-based strategy against lifestyle maladies. Food Sci Nutr 7:3378–3390 31762991 10.1002/fsn3.1218 PMC6848808

[8] Coutino-Rodriguez R, Hernandez-Cruz P, Gillis-Rios H (2001) Lectins in fruits having gastro-intestinal activity and their partic-ipation in the hemagglutinating property of Escherichia Coli 0157. Archives Medicial Res 32:251–259 10.1016/s0188-4409(01)00287-9 11440778

[9] El-Hawary SSE, El Zalabani SM, Ibrahim SNM, Wahba MA, Badawy FAE, Mahdy SA, Yasri NES, Sobeh A M (2019) Phenolic con-stituents of Chrysophyllum oliviforme L. leafdown-regulate TGF-β expression and ameliorate CCl4-induced liver fibrosis: evidence from in vivo and in Silico studies. Antioxid (Basel) 15(12):646 10.3390/antiox8120646 PMC6943707 31847463

[10] Escalona-Arranz JC, Péres-Roses R, Urdaneta-Laffita I, Camacho-Pozo MI, Rodríguez-Amado J, Licea-Jiménez I (2010) Antimicrobial activity of extracts from Tamarindus indica L. leaves. Pharmacognosy Magazine 6(23):242 20931087 10.4103/0973-1296.66944 PMC2950390

[11] Nakchat O, Nalinratana N, Meksuriyen D, Pongsamart S (2014) Tamarind seed coat extract restores reactive oxygen species through Attenuation of glutathione level and antioxidant enzyme expression in human skin fibroblasts in response to oxidative stress. Asian Pac J Trop Biomed 4(5):379–385 25182723 10.12980/APJTB.4.2014C806 PMC3985053

[12] Vasant RA, Narasimhacharya AV (2012) Ameliorative effect of tamarind leaf on fluoride-induced metabolic alterations. Environ Health Prev Med 17(6):484–493 22438201 10.1007/s12199-012-0277-7 PMC3493631

[13] Yanez E, Zacarias I, Aguayo M, Vasquez M, Guzman E (1995) Nutritive value evaluation rats of new cultivars of common beans (Phaseolus vulgaris) released on Chile. Plant Foods Hum Nutr 47:301–307 8577647 10.1007/BF01088267

[14] Gupta C, Prakash D, Gupta S (2014) Studies on the antimicrobial activity of tamarind (Tamarindus indica) and its potential as food bio-preservative. Int Food Res J 21(6):2437–2441

[15] Khamis G, Hassan M, Morsy M, Ibrahim MA, El Badawy AE, Asmaa A, Azouz AA, Galal M (2020) Innovative application of helium-neon laser: enhancing the germination of Adansonia digitata and evaluating the hepatoprotective activities in mice. Environ Sci Pollut Res 27:26520–26531 10.1007/s11356-020-09036-0 32367237

[16] Perveen R, Jamil Y, AshrafM, Ali Q, Iqbal M, AhmadMR (2011) He-Ne laser-induced improvement in biochemical, physiological, growth and yield characteristics in sunflower (Helianthus annuus L). Photochem Photobiol 87:1453–1463 21790619 10.1111/j.1751-1097.2011.00974.x

[17] Hassan M, Shaaban SA, El Ziat RA et al (2024) Laser-induced changes in the gene expression, growth, and development of *Gladiolus grandiflorus* cv. White prosperity. Sci Rep 14:6257 38491044 10.1038/s41598-024-56430-6 PMC10943131

[18] Salehinia S, Mirsaeedghazi H, Khashehchi M (2021) The effect of laser on the efficiency of membrane clarification of pomegranate juice. J Food Sci Technol 58(5):1682–1692 33897007 10.1007/s13197-020-04678-x PMC8021681

[19] Okla MK, El-Tayeb MA, Qahtan AA, Abdel-Maksoud MA, Elbadawi YB, Alaskary MK, Balkhyour MA, Hassan AHA, Abd, Elgawad (2021) H. Laser light treatment of seeds for improving the biomass photosynthesis, chemical composition and biological activities of Lemongrass sprouts. Agronomy 11:478

[20] Dinani HJ, Eslami P, Mortazaeinezhad F, Taheri R, Ghahrizjani RT (2019) Effects of laser radiation on the growth indicators of kelussia odoratissima mozaff. Medical plant. IEEE Photonics North (PN), pp 1–8

[21] Leng LY, Nadzri N, Shaari AR, Norawanis AR, Khor CY (2017) Antioxidant capacity and total phenolic content of fresh, Oven-Dried and Stir-Fried tamarind leaves. Curr Res Nutr Food Sci 5(3)

[22] Massada Y (1976) Analysis of essential oil by gas chromatography and spectrometry. John Wiley & Sons Inc, NewYork

[23] Velmurugan G, Anand SP (2017). GC-MS analysis of bioactive compounds on ethanolic leaf extract of phyllodium, vol 9. IJPPR

[24] Mulla SI, Wang H, Sun Q, Hu A, Yu CP (2016a) Characterization of triclosan metabolism in Sphingomonas sp. strain YL-JM2C. Sci Rep 6:21965 26912101 10.1038/srep21965 PMC4766416

[25] Gaetani R, Lacotte V, Dufour V, Clavel A, Duport G, Gaget K, Calevro F, Da Silva P, Heddi A, Vincent D, Masenelli B (2021) Sustainable laser-based technology for insect pest control. Sci Rep 11(1):11068 34040124 10.1038/s41598-021-90782-7 PMC8155209

[26] Gorinstein S, Medina Vargas OJ, Jaramillo NO, Salas IA, Ayala ALM, Arancibia-Avila P et al (2007) The total polyphenols and the antioxidant potentials of some selected cereals and pseudocereals. Eur Food Res Technol 225:321–328

[27] Boly R, Lamkami T, Lompo M, Dubois J, Guissou I (2016) DPPH free radical scavenging activity of two extracts from Agelanthus Dodoneifolius (Loranthaceae) leaves. Int J Toxicol Pharmacol Res 8(1):29–34

[28] Meher B, Dash DK (2013) Antioxidant and antimicrobial properties of Tamarindus indica L. Int J Phytomedicine 5(3):322–329

[29] Saacson T, Damasceno CMB, Saravanan RS, He YH, Catalµ C, Saladi M, Rose JKC (2006) Nat Protoc 1:769–774 10.1038/nprot.2006.102 17406306

[30] Alexeree SMI, ElZorkany H, Abdel-Salam Z, Harith MA (2021) A novel synthesis of a chlorophyll b-gold nanoconjugate used for enhancing photodynamic therapy: in vitro study. Photodiagn Photodyn Ther 35:102444 10.1016/j.pdpdt.2021.102444 34284147

[31] Abirami P, Rajendran A (2011) GC-MS determination of bioactive compounds of Indigofera aspalathoides. J Nat Prod Plant Resour 1(4):126–130

[32] Chen Y-P, Yue M, Wang X-L (2005) Influence of He–Ne laser irradiation on seeds thermodynamic parameters and seedlings growth of Isatis indogotica. Plant Sci 168(3):601–606

[33] Ullah R et al (2018) GC-MS profile of bioactive compounds from medicinally important Periploca hydaspidis. Pak J Pharm Sci 31(5):1967–1973 30150196

[34] Mozaffarian D, Cao H, King IB, Lemaitre RN, Song X, Siscovick DS, Hotamisligil GS (2010) Trans-palmitoleic acid, metabolic risk factors, and new-onset diabetes in U.S. Adults: a cohort study. Ann Intern Med 153(12):790–799 21173413 10.1059/0003-4819-153-12-201012210-00005 PMC3056495

[35] Zhang X, Wang Y (2023) The role of the novel lipokine palmitoleic acid in health and disease. Trends Endocrinol Metabolism 34(3):230–241

[36] Li Y et al (2024) Therapeutic potential of palmitoleic acid in non-alcoholic fatty liver disease. Biochimie 210:15–25

[37] Kumar NG, Contaifer D, Madurantakam P, Carbone S, Price ET, Van Tassell B, Brophy DF, Wijesinghe DS (2019) Dietary bioactive fatty acids as modulators of immune function: implications on human health. Nutrients 11(12):2974 31817430 10.3390/nu11122974 PMC6950193

[38] Agati G, Azzarello E, Pollastri S, Tattini M (2012) Flavonoids as antioxidants in plants: location and functional significance. Plant Sci 196:67–76 23017900 10.1016/j.plantsci.2012.07.014

[39] Osman YAH, El-Tobgy KMK, El-Sherbini ESA (2009) Effect of laser radiation treatments on growth, yield and chemical constituents of fennel and coriander plants. J Appl Sci Res 5:244–252

[40] Dutta Gupta S, Kumar A, Agarwal A (2019) Impact of light-emitting diodes (LEDs) on the growth and morphogenesis of encapsulated shoot buds of Curculigo orchioides Gaertn an endangered medicinal herb. Acta Physiol Plant 41:1–12

[41] Mousa FM, Ali MM, Abdel-Halim AHAH, Khamis G, Morsy M, Ghanem HM (2023) Assessment the effect of He-Ne laser treatment of *Balanites aegyptiaca* seeds on the amelioration of active constituents, antioxidant capacity, and anticancer impact *in vitro*. Egypt Pharm J 22:1

[42] Almuhayawi MS (2021) Laser light as a promising approach to improve the nutritional value, antioxidant capacity and anti-inflammatory activity of flavonoid-rich buckwheat sprouts. Food Chem 345:128788Hassan, A.H.A., Abdel-Mawgoud, M., Khamis, G., Selim, S., Al Jaouni, S. K., AbdElgawad, H. 33340896 10.1016/j.foodchem.2020.128788

[43] Perveen R, Ali Q, Ashraf M, Al-Qurainy F, Jamil Y, Raza Ahmad M (2010) Effects of different doses of low power continuous wave He–Ne laser radiation on some seed thermodynamic and germination parameters, and potential enzymes involved in seed germination of sunflower (Helianthus annuus L). Photochem Photobiol 86(5):1050–1055 20670360 10.1111/j.1751-1097.2010.00782.x

[44] Hasan M, Hanafiah MM, Alhilfy IHH, Aeyad TZ (2021) Comparison of the effects of two laser photobiomodulation techniques on bio-physical properties of Zea mays L. seeds. Peer J 15:9:e10614 10.7717/peerj.10614 PMC7812920 33520446

[45] Abou-Dahab MAD et al (2019) In vitro laser radiation induces mutation and growth in Eustoma grandiforum plant. Bull Natl Res Centre 43:3

[46] Torat SA et al (2021) Red laser-mediated alterations in seed germination, growth, pigments and withanolide content of Ashwagandha [Withania somnifera (L.) Dunal]. J Photochem Photobiol B: Biol 216:112144 10.1016/j.jphotobiol.2021.112144 33556702

[47] Shehata AM, Azoz S, Khaled KA, Hassan M, Tawfic G, Fahmy MA (2023) Physiological, anatomical, chemical, and genetic responses of two snap bean (Phaseolus vulgaris L.) varieties to invitro optical Bio-stimulation induction. Sci J Agricultural Sci 5(1):49–72

[48] Abdani NA, Mortazaeinezhad F, Taheri R (2018) Seed germination of medicinal Sage is afected by gibberellic acid, magnetic feld and laser irradiation. Electromagn Biol Med 37(1):50–56 29308934 10.1080/15368378.2017.1336100

[49] Jin D et al (2023) Efect of red and blue light on cucumber seedlings grown in a plant factory. Horticulturae 9(2):124

[50] Aghaebrahimi Z, Sabaghzadeh J, Soudi S et al (2024) Simultaneous effect of medicinal plants as natural photosensitizers and low-level laser on photodynamic inactivation. Lasers Med Sci 39:95 38538952 10.1007/s10103-024-04037-8

[51] Hassan M, Hanafiah MM, Taha ZA, Alhilfy IHH, Said MNM (2020) Laser irradiation effects at different wavelengths on phenology and yield components of pretreated maize seed. Applied Sciences 10(3):1189

[52] Heinemann D, Zabic M, Terakawa M (2022) Boch, Laser-based molecular delivery and its applications in plant science. Plant Methods 18:82 35690858 10.1186/s13007-022-00908-9 PMC9188231

[53] Altemimi A, Lakhssassi N, Baharlouei A, Watson DG, Lightfoot DA (2017) Phytochemicals: extraction, isolation, and identification of bioactive compounds from plant extracts. Plants 6(4):42 10.3390/plants6040042 PMC5750618 28937585

[54] Alexeree Sh MI, Book (2025) Elsevier Inc. 459–470

[55] Youssef D, Alexeree S, Abdel-Harith M (2023) Biospeckle photography for quantitative evaluation of photodynamic therapy-mediated phthalocyanine-gold nanoconjugates. Frontiers in Optics + Laser science 2023 (FiO, LS). Technical Digest Series. Optica Publishing Group. paper FD6.5.

[56] Nowruzi B, Aljashamy H, Firuzabad MZ (2023) Study of pesticidal activity of bioactive compounds of Desmonostoc alborizicum in improving the antioxidative activity of Glycine max to Cowpea aphid. Arthropod-Plant Interact 17:811–824

[57] Aires-Fernandes M, Costa B, Rochetti do Amaral R, Mussagy S, Santos-Ebinuma CU, Primo VC, F.L (2022) Development of biotechnological photosensitizers for photodynamic therapy: Cancer research and Treatment—From benchtop to clinical practice. Molecules 27:6848 36296441 10.3390/molecules27206848 PMC9609562

[58] Alexeree SMI, Harith MA (2023) Monitoring the cytotoxic effect of novel nanoconjugates during and after in-vivo photodynamic therapy. AIP Conference Proceedings 2620:060001

